# Elucidating the cancer-specific genetic alteration spectrum of glioblastoma derived cell lines from whole exome and RNA sequencing

**DOI:** 10.18632/oncotarget.6171

**Published:** 2015-10-19

**Authors:** Vikas Patil, Jagriti Pal, Kumaravel Somasundaram

**Affiliations:** ^1^ Department of Microbiology and Cell Biology, Indian Institute of Science, Bangalore, India

**Keywords:** glioblastoma, exome & RNA sequencing, cancer-specific mutations, gene fusions, RNA editing

## Abstract

Cell lines derived from tumor tissues have been used as a valuable system to study gene regulation and cancer development. Comprehensive characterization of the genetic background of cell lines could provide clues on novel genes responsible for carcinogenesis and help in choosing cell lines for particular studies. Here, we have carried out whole exome and RNA sequencing of commonly used glioblastoma (GBM) cell lines (U87, T98G, LN229, U343, U373 and LN18) to unearth single nucleotide variations (SNVs), indels, differential gene expression, gene fusions and RNA editing events. We obtained an average of 41,071 SNVs out of which 1,594 (3.88%) were potentially cancer-specific. The cell lines showed frequent SNVs and indels in some of the genes that are known to be altered in GBM- EGFR, TP53, PTEN, SPTA1 and NF1. Chromatin modifying genes- ATRX, MLL3, MLL4, SETD2 and SRCAP also showed alterations. While no cell line carried IDH1 mutations, five cell lines showed hTERT promoter activating mutations with a concomitant increase in hTERT transcript levels. Five significant gene fusions were found of which NUP93-CYB5B was validated. An average of 18,949 RNA editing events was also obtained. Thus we have generated a comprehensive catalogue of genetic alterations for six GBM cell lines.

## INTRODUCTION

Grade IV glioma or glioblastoma multiforme (GBM) is the most common and aggressive primary brain tumor. It accounts for 20% of all intracranial tumors [1] and comprises of neoplastic glial cells called astrocytes. GBM can arise *via* the *de novo* pathway without clinical or histologic evidence of a less malignant precursor lesion (primary GBM) or *via* the progressive pathway through development from a low-grade astrocytoma (secondary GBM) [2]. With the current mode of treatment of surgery along with temozolomide chemotherapy and radiotherapy, the median survival achieved till today is only 14.6 months [3].

Malignant tumors arise when genomic lesions accumulate within cells that disrupt normal cellular pathways ultimately giving them a survival advantage leading to tumor initiation, growth and metastasis. Each tumor carries a combination of genetic alterations that determine cancer prognosis and response to therapy. GBM tumors show significant amount of proliferation, invasion, angiogenesis and necrosis and is treatment refractory. In the past two decades, focused studies on candidate genes show various genetic alterations typical to GBM, e.g., TP53 mutation and loss, EGFR amplification and mutation, INK4a/ARF mutation, MDM 2/4 amplification or overexpression, PTEN mutation and loss of heterozygosity (LOH) in chromosome 10p and 10q [4, 5]. In recent times, the advent of next generation sequencing (NGS) technologies has paved the path to analysis of entire cancer genome [6, 7]. Whole exome sequencing (WES) and RNA sequencing (RNA-seq) are two techniques that can provide information for the functionally relevant part of the genome at increased coverage and reduced cost. Recently, two independent groups have carried out exome and RNA-seq analysis of GBM tissue samples and have found out various novel genetic alterations which may play important role in GBM development and progression [8, 9].

Established cell lines from tumors play an important role as *in vitro* model to study various aspects of tumor development and progression. A comprehensive understanding of the genomic make-up of the cell lines will provide us with information regarding the alteration status of the genes present in the cell lines thus giving us an opportunity to choose the cell lines appropriately for particular studies. There have been three studies which characterized glioma derived cell lines using next generation sequencing [10-12]. However, these studies have carried out either whole genome or whole exome or RNA sequencing. Here, we have carried out an elaborate study to comprehensively characterize six GBM cell lines that are most commonly used. Both whole exome sequencing and whole RNA sequencing was carried out and in-depth analysis was performed to find out single nucleotide variations (SNVs), insertions/deletions (indels), transcriptional changes, gene fusions and RNA editing events. To our knowledge, this study is the first time an in-depth characterization of the genomic alterations present in these cell lines have been carried out and we believe that this information will be highly useful to the scientific community.

## RESULTS

### WES and RNA-seq statistics and quality assessment

Genomic DNA from six GBM cell lines (U87, T98G, U343, LN229, U373, and LN18) was subjected to TruSeq exome capture and sequenced in Illumina HiScanSQ platform (100 bp paired-end sequencing). Data analysis was carried out as given in Materials and Methods section. The raw reads were aligned to human reference genome hg19 and the initial quality statistics were assessed (Table 1). For each cell line, on an average 52,629,690 reads passed quality criteria of Qscore (Phred quality score) ≥ 30. While the average percentage of reads that mapped to hg19 was 98.48% across all cell lines, the average percentage of properly paired reads was 97.56%. The targeted region (genomic regions covered by Illumina's exome capture kit) covered by the quality passed reads was 99.68%. We obtained an average coverage of ∼36.31X which is suitable for calling variants with confidence as per Illumina guidelines [13].

**Table 1:** Whole exome and whole RNA sequencing statistics and quality assessment of glioma cell lines

Cell line	U87	T98G	LN229	U343	U373	LN18	Average
**I - Whole Exome Sequencing**							
QC-passed reads*	41,937,382	56,365,156	59,066,920	60,402,106	48,108,386	49,898,190	**52,629,690**
**Reads mapped (%)**	98.53	98.22	98.43	98.62	98.89	98.18	**98.48**
**Properly paired reads (%)**	97.97	97.41	97.44	97.69	98.33	96.52	**97.56**
**Target regions covered (%)**	99.50	99.80	99.70	99.50	99.80	99.80	**99.68**
**Average coverage (X) for whole exome**	29.66	39.61	41.53	37.13	35.78	34.13	**36.13**
**II - Whole RNA Sequencing**							
**QC-passed reads**	33,796,760	49,895,568	42,609,412	92,046,396	47,085,884	97,773,184	**60,534,534**
**Reads mapped (%)**	89.63	94.36	96.53	96.87	96.73	94.17	**94.72**

* QC-passed reads= reads having Phred quality score ≥ 30

Similarly, total RNA from the above six cell lines was subjected to RNA-seq in Illumina HiScanSQ platform (50 bp paired-end sequencing). The average number of reads obtained from each cell line came to be around 60,534,534. The percentage of reads obtained from the sequencing that mapped to the human reference genome hg19 was 94.72% (Table 1). Given the fact that a minimum number of 25 million reads per sample is sufficient for RNA-seq data analyses, we found our samples with more than 33 million reads suitable for our study [14].

### Identification of single nucleotide variations (SNVs) and indels

The variant calls were generated using GATK tool following the filtering criteria: 1) all SNVs detected should be restricted to the 62 Mb region targeted by Illumina Truseq exome capture kit, 2) bases having quality score above 30 should be considered and 3) minimum 6 reads carrying variant bases should be present to be considered as an SNV. We obtained an average number of 41,071 SNVs, out of which 39,652 SNVs were present in the single nucleotide polymorphism database (dbSNP) 137 build while 1,156 were novel SNVs (Table 2). Further, we found an average of 20,768 homozygous and 20,302 heterozygous SNVs. The average transition vs transversion (Ti/Tv) ratio was 2.42 (Table 2) [10].

**Table 2:** Single nucleotide variation and indel classification and quantification

Cell line	U87	T98G	LN229	U343	U373	LN18	Average
**I. Single Nucleotide Variations (SNVs)**							
**Total variants detected**	**38,515**	**42,423**	**42,788**	**43,582**	**40,095**	**39,020**	**41,071**
Variants present in dbSNP*	37,545	41,029	41,196	42,001	38,915	37,224	39,652
Novel variants**	970	1,394	1,592	1,581	1,180	1,796	1,156
**Homozygous variants**	19,710	20,762	21,293	17,104	22,175	23,566	20,768
**Heterozygous variants**	18,805	21,661	21,495	26,478	17,920	15,454	20,302
**Ti/Tv Ratio**	2.49	2.42	2.38	2.44	2.41	2.38	2.42
**II. Indels**							
**Total indels detected**	**3,780**	**3,968**	**4,000**	**4,151**	**3,779**	**3,672**	**3,892**
Indels present in dbSNP*	3,528	3,693	3,712	3,831	3,515	3,390	3,612
Novel indels*	255	275	288	320	264	282	280
**Homozygous indels**	1,963	2,045	2,148	1,730	2,144	2,180	2,035
**Heterozygous indels**	1,817	1,923	1,852	2,421	1,635	1,492	1,857

* dbSNP refers to the Single Nucleotide Polymorphism database.

** In comparison with dbSNP Build 137.

Small insertions-deletions (indels) were identified with GATK tool using similar filtration criteria as used for SNVs. The list of indels found from WES is given in Supplementary Table 1. Indels were observed to range in size between −49 to +29 bases (Supplementary Figure S1A) and were detected at a proportion of 10 to 12 % of that of SNVs (Table 2) [15]. The average number of indels detected was 3,892 out of which 3,612 were present in dbSNP build 137 while 280 were novel (Table 2). Although the indels distribution followed power law distribution, there was small deviation at 4-base indels (Supplementary Figure S1A) [16]. Interestingly, when the indel distribution in the coding region alone was looked into, there was an enrichment of indels of size equal to multiples of three bases (Supplementary Figure S1B).

Next, we classified the SNVs and indels using Oncotator [17], according to their location in the genome and also the type of changes the alteration will bring to the protein (Supplementary Figure S1C, D, E, F). While SNVs were found in equal proportion between non-coding and protein coding regions, a tenfold more occurrence of indels in non-coding region compared to protein coding regions was found (Supplementary Figure S1C and D). We found a high occurrence of SNVs in the form of missense and silent mutations compared to a very low occurrence in the form of nonsense, nonstop, splice site and translation start site mutation (Supplementary Figure S1E). Indels resulted in frame-shift, in-frame and splice site changes at a higher frequency, but it was found rarely in translational start sites. (Supplementary Figure S1F).

### Comparison of SNVs from exome data to catalogue of somatic mutations in cancer (COSMIC) and cancer cell line encyclopedia (CCLE) databases

COSMIC database contains most comprehensive resource of genetic alterations occurring in a large number of human cancer tissue and cancer derived cell lines [18]. CCLE project undertaken by Broad Institute provides information for mutations, copy number variations (CNVs) and mRNA expression in a large panel of cell lines [19]. To test the robustness of in-house WES data, we compared SNVs detected in this study with COSMIC and CCLE databases. SNV information was available for four of the six cell lines we have studied (U87, T98G, LN229 and LN18) in COSMIC and CCLE databases. We found that our data had an average concordance of 65.3% with COSMIC and 74.74% with CCLE databases (Supplementary Figure S2). A more in-depth comparison of cancer specific mutations between our finding and COSMIC database is described later (Figures 3 and 4).

### Detection of cancer-specific SNVs from WES data

**Figure 1:**
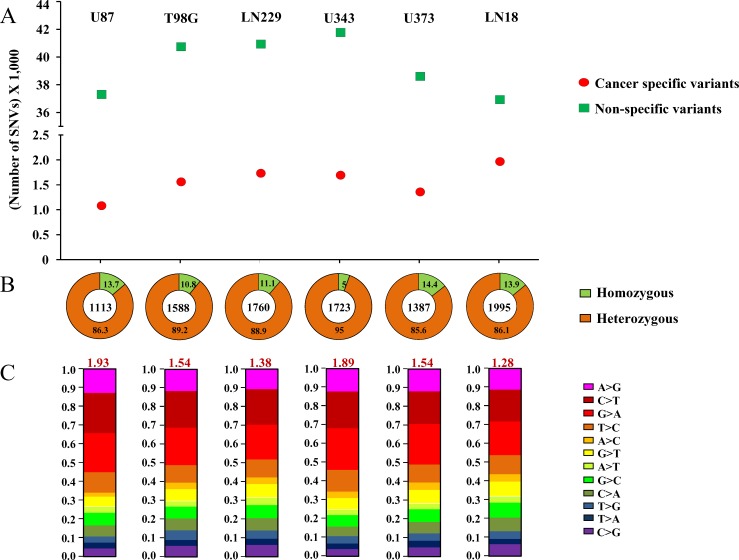
Cancer-specific mutation quality statistics **A.** Number of Cancer-specific *versus* non-specific SNVs. **B.**. Percentage of homozygous *versus* heterozygous changes. The numbers in each quadrant represent the percentage, and the number in the center of the circle represents the total number of cancer-specific mutations. C. Ti/Tv changes for each cell line's cancer-specific SNVs. Each type of base change has been given by different color codes and the overall Ti/TV ratio has been given in red font at the top of each bar-plot. For **A.**, **B.** and **C.**, plot for each cell line has been given in one column and the name of the cell line has been specified at the top.

Due to unavailability of matched normal samples and also to eliminate the mutations which may have no role in cancer development and progression, a stringent filtration criteria was followed as described in Materials and Methods section and the SNVs were divided into cancer-specific and non-specific types (Supplementary Figure S9). The list of cancer-specific SNVs is given in Supplementary Table 2. The average percentage of cancer-specific SNVs for each cell line was found to be 3.88% of the total number of SNVs obtained. The average number of cancer-specific and non-specific SNVs across the cell lines came to be approximately 1,595 and 39,476 respectively (Figure 1A). The number of non-specific SNVs was quite variable for each cell line, although the number of cancer-specific SNVs showed more or less equal frequencies. The cell line LN18 showed a higher cancer-specific SNV rate compared to its relatively lower non-specific SNVs. Previous reports identified that cell lines with microsatellite instability (MSI), which arises due to mutation in mismatch repair (MMR) genes, harbored several fold higher cancer-specific mutations compared to those cell lines without MSI [11]. In our study, the frequency of cancer-specific mutations in all the six cell lines was in a much lower range (1595 ± 310), same as that of cell lines without MSI as previously reported [11]. Concomitantly, we also found that none of cell lines studied here harbored mutation in MMR genes (MSH2, MSH3, MSH6, MLH1, PMS2, MSH4, MSH5, MLH3, PMS1 and PMS2L3). This is expected because microsatellite instability is a rare phenomenon in GBM [20].

**Figure 2:**
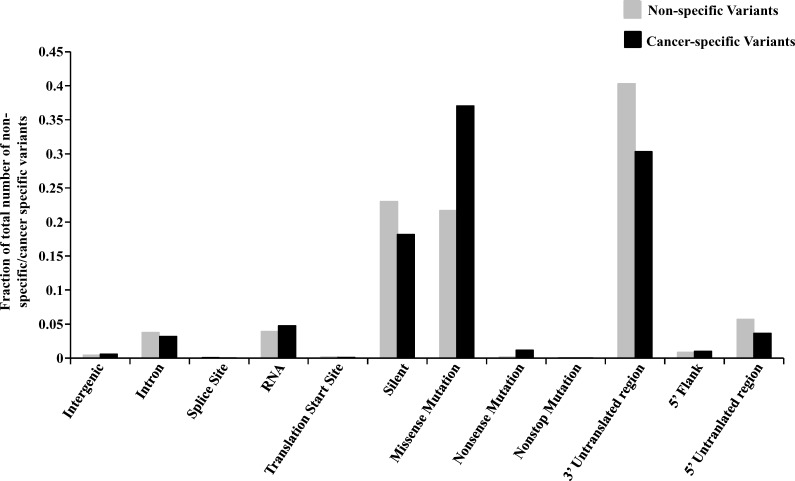
Functional classification of cancer-specific *versus* non-specific variants The variants have been plotted as a fraction of the total number of cancer-specific (CS) or non-specific (NS) variants. Fraction of CS and NS variants have been plotted for each functional type according to their location in the genome i.e. inter-genic region, intron, 5′ flank, 5′ UTR, 3′ UTR, RNA, splice site, translation initiation site and coding region which has been further subdivided into the type of change the alteration will bring to the protein (missense, nonsense, non-stop and silent).

The percentages of homozygous and heterozygous SNVs for cancer-specific changes have been represented (Figure 1B). The ratio of the homozygous to the heterozygous variants was around 1:8. Somatic mutations being sporadic and random in nature, the probability of both alleles being mutated similarly is rare which is why cancer-specific SNVs show a very high proportion of heterozygous changes as compared to homozygous changes. The proportion of each type of base changes for the cancer-specific SNVs for each cell line has been given (Figure 1C). The average Ti/Tv ratio for each cell line came to be around 1.66 (ranging from 1.28 to 1.93), in a similar range as reported before [11]. The proportion of each functional type of SNVs, depending upon their genomic location or the consequence that the SNV can bring about in the gene product i.e. changes in exonic regions, has been plotted for both types of variants (Figure 2). It was observed that the proportion of cancer-specific SNVs is higher in regions where nucleotide changes have a deleterious consequence. This includes the functional classes ‘missense mutation’ and ‘nonsense mutation’, as previously reported, as compared to other regions where non-specific SNV proportion was observed to be more [11].

### Comparison of indels and cancer-specific SNVs with TCGA, COSMIC and CCLE databases

**Figure 3:**
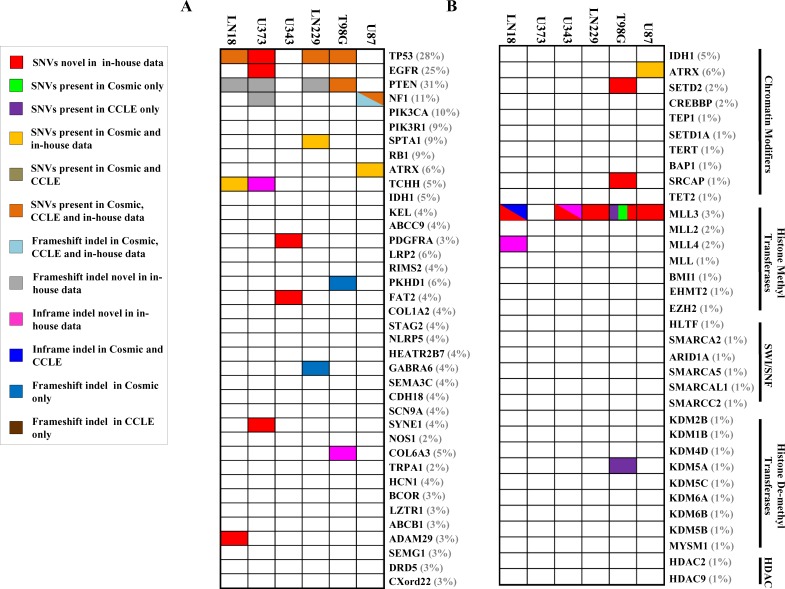
Mutation spectrum for most commonly mutated genes and chromatin modifying genes in each cell line **A.** SNV and indel status of genes (*n* = 38) in cell lines that are frequently altered in GBM [8, 9]. **B.** SNV and indel status of frequently altered chromatin modifying genes (*n* = 34) [9]. The percentage at which each gene is mutated in TCGA GBM tissue [9] has been provided in brackets after each gene name. **C**omparison with COSMIC & CCLE databases for the same genes **A.** and **B.** is also shown. The dual/triple colors indicate that the gene harbors two/three types of mutations in the particular cell line respectively.

**Figure 4:**
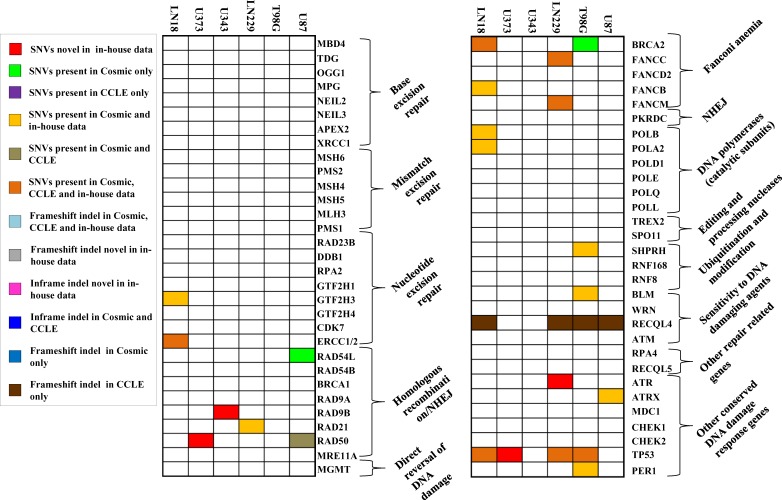
Mutation spectrum for DNA repair genes in each cell line SNV and indel status of genes (*n* = 61) in cell lines that are altered in GBM [9]. Comparison with COSMIC & CCLE databases for the same genes is also shown.

We investigated the overlap between mutations (indels and cancer-specific SNVs) identified in the six cell lines and the significantly mutated genes in GBM tumor samples as per large scale exome sequencing [8, 9]. Figure 3A shows the map of occurrence of alterations in these genes for the six cell lines studied. Out of 38 genes tested, we found mutations in 12 genes in one or more cell lines from WES. As previously reported, the genes TP53, PTEN and EGFR are mutated in more than 20 % of TCGA GBM tumor tissue samples [8, 9]. We found PTEN and TP53 to be mutated in 4 out of 6 cell lines, while EGFR was mutated in one cell line only (Figure 3A). Among the genes that are mutated between 5 to 20% in TCGA GBM tumor samples [8, 9], we found mutations in NF1, SPTA1, TCHH and ATRX genes, although, the other genes like PIK3R1, PIK3CA, RB1, IDH1 and KEL were not mutated in any cell line (Figure 3A). Upon analyses of the status of a set of 34 chromatin modifying genes [9], we found mutations in ATRX, SETD2, SRCAP, MLL3 and MLL4, genes (Figure 3B). Investigation of the mutation status of DNA repair genes that were mutated in TCGA study revealed that out of 61 genes that were analysed, 17 genes harbored SNVs in one or more cell lines (Figure 4). No indels were observed in the DNA repair genes. No mutation was observed in any of the genes related to base excision repair (BER) and also, in MMR genes (Figure 4).

We also investigated the overlap between indels and cancer-specific SNVs with that of COSMIC and CCLE databases for four cell lines which are common between these data sets [18] (Figures 3 and 4). A total of 32 mutations were uncovered in 129 genes tested from WES in U87, T98G, LN229 and LN18 (Figures 3 and 4) out of which 21 mutations were reported either in COSMIC or CCLE or both datasets giving a concordance of 65.6%, and the rest 11 mutations were found novel from WES data (Supplementary Table S3). Further, 13 novel mutations were also observed in the remaining two cell lines, U343 and U373 (Figures 3 and 4; Supplementary Table 3). From the 24 novel mutations found across all six cell lines, 8 were selected for validation by Sanger sequencing out of which 7 could be validated (Supplementary Figure S3).

### Functional importance of cancer-specific SNVs

The cancer-specific SNVs obtained in this study might have potential carcinogenic consequence as these changes are not observed in multiple normal human sequence databases. Out of 129 genes as in Figures 3 and 4, 30 genes were seen to harbor non-synonymous cancer-specific SNVs in one or more cell lines. We used PolyPhen-2 and SIFT tools on the above 30 genes to predict whether a particular cancer-specific SNV can alter the function of the protein significantly [21, 22]. Mutations that were predicted by both tools to be deleterious or damaging were considered. Thirty percent of genes (*n* = 9) were found to contain SNVs that attribute a significant change in protein function (Supplementary Table S4). The functional significance of the SNVs present in NF1, MLL3, BRCA2, ATR, ERCC2 and TP53 was further investigated. The protein domain structure and the positions mutated in each protein is given (Supplementary Figure S4). NF1 is a negative regulator of RAS signaling pathway. It has a RAS-GAP domain (1174-1535 amino acid positions) which functions to convert GTP in activated RAS to GDP, thus inactivating RAS. Recurrent mutations are found in GBM tumor in NF1 RAS-GAP domain [9]. NF1 mutation in U87 occurs in this domain where lysine residue at 1444 position gets converted to methionine which could lead to abrogation of GTPase activity of NF1 [23]. The tumor suppressor gene MLL3 is mutated in three positions, G892R, Y987H and C988F, in different cell lines (Supplementary Table 4). Of the three positions, Y987H and C988F are reported in COSMIC database [18]. MLL3 is a nuclear protein comprising of 6 PHD finger domains, one of each of HMG, FYRN, FYRC and SET domains [24] and it functions as a histone methyl transferase. In cancer scenario, it has been observed that enrichment of mutations is present in the PHD, FYNC, FYRC and SET domains [25]. The mutations, Y987H and C988F, found in the cell lines are present in PHD finger domain of MLL3. PHD finger domains are required for proper histone methylation [25] and mutation in this domain will significantly abrogate MLL3 function. LN18 cell line was seen to harbor a deleterious mutation in BRCA2 (N856Y). BRCA2 interacts with various proteins like RAD51, PALB2, NPM1, PCID2 and DSS1 and is mainly involved in DNA double strand break repair. The above mutation in BRCA2 is present in the region which is involved in binding to NPM1 [26]. ATR is a kinase that gets activated when DNA damage occurs within the cell. LN229 cell line was seen to harbor G2375R mutation in the kinase domain (2321-2567 amino acid positions) of ATR which might severely abrogate its function [27]. ERCC2, mutated in LN18 (R690W), is a nuclear protein involved in the repair of damaged DNA through nucleotide excision repair pathway. This particular mutation is present in the nuclear localization signal sequence (682-696 amino acid positions) of ERCC2 and this will hamper the targeting of ERCC2 to the nucleus [28]. The tumor suppressor gene, TP53, is involved in the regulation of apoptosis, genome stability and angiogenesis and it is found to be mutated in almost all types of cancer. In this study, 4 out of 6 cell lines was found to be mutant for TP53 (T98G, LN229, U373 and LN18) where the mutations were seen to be present in the DNA binding domain (95-289 amino acids), thus leading to significant functional alteration in the protein. Since TP53 is a transcriptional regulator, its mutation will have a direct effect on the transcriptome of the cell.

We next sought to find out the alteration in the transcriptome in p53 mutant *versus* p53 wild-type cell lines from RNA-seq data. The expression levels of 227 genes were found to be altered in p53 mutant cell lines as compared to p53 wild type ones (Supplementary Table 5). These 227 genes were subjected to analysis using DAVID tool to find out pathways enriching in p53 mutant conditions (Supplementary Figure S5) [29, 30]. p53 regulates multiple processes within the cell which include cell differentiation [31-33], neuron development [34-36], cell adhesion [37-40], cell proliferation and apoptosis [41-47]. All these biological processes were seen to be significantly enriched when above 227 genes were subjected for pathway enrichment in DAVID Gene Ontology (GO) analysis (Supplementary Figure S5).

**Figure 5:**
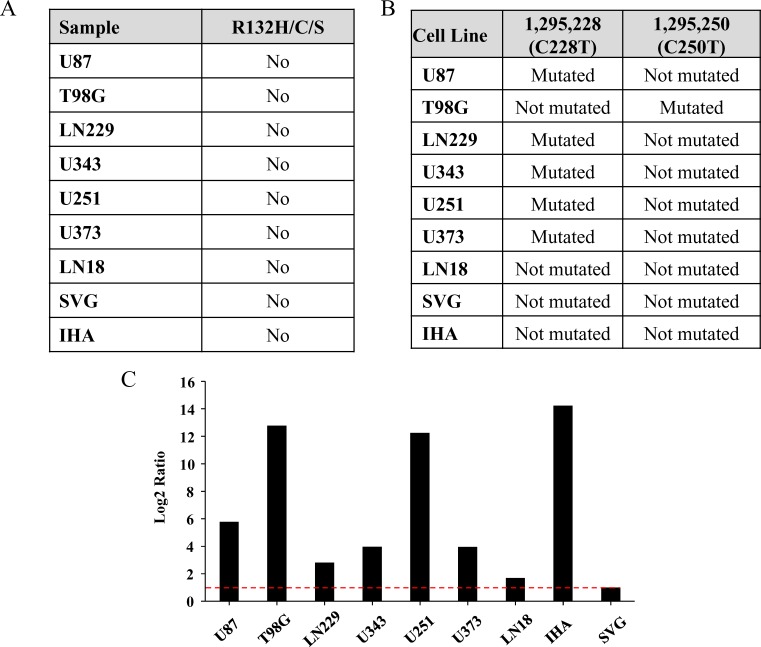
Mutation status of IDH1 and hTERT promoter in the cell lines **A.** Mutation status of IDH1 position R132H/C/S in each cell line. **B.** Presence or absence of C228T or C250T mutations in the promoter region of hTERT in each cell line. **C.** Real-time PCR quantification of hTERT mRNA in different cell lines has been given. The mRNA level for SVG has been normalized to 1 and the levels for other cell lines have been given in comparison to that of SVG. Red line indicates 1 in log2 scale.

We next investigated the mutation status of isocitrate dehydrogenase genes (IDH1 and IDH2) in the cell lines. Mutation in IDH1 is observed in ∼80% of grade 2 and 3 gliomas and secondary GBM [48, 49]. The typical mutation that occurs in gliomas is R132H mutation, although R132C or R132S is also seen at much lower percentages [50]. Paralog of IDH1, i.e., IDH2 mutated in the position R172 also show similar effect although frequency of mutation in IDH2 is rare [51]. No mutations in IDH1or IDH2 were obtained from WES data in the cell lines tested. IDH1 mutation status was further validated through Sanger Sequencing, which included an additional three cell lines - U251, SVG and immortalized human astrocytes (IHA) (Figure 5A; Supplementary Figure S6A). Thus the cell lines used are derived from primary GBM patients.

Telomerase activation caused by promoter mutation in glioma has been reported recently [52-54]. There are two mutations, C > 1,295,228 > T and C > 1,295,250 > T reported in the promoter region of hTERT gene. Fifty five percent of GBM tumor samples harbor hTERT promoter mutation where the above two mutations are mutually exclusive [54]. Since the exome enrichment method used does not capture the hTERT promoter region, we carried out Sanger sequencing to detect hTERT promoter mutations. U87, LN229, U343, U251, and U373 cell lines showed C228T whereas; T98G showed C250T promoter activating mutations (Figure 5B; Supplementary Figure S6B). GBM cell line LN18 and the immortalized normal astrocytes SVG and IHA showed no mutation in hTERT promoter. RT-qPCR for hTERT mRNA revealed increased levels in cell lines carrying hTERT promoter mutations in GBM cell lines (U87, LN229, U343, U251, U373 and T98G) and IHA in comparison to wild type cell lines (LN18 and SVG) (Figure 5C). IHA, harboring wild-type hTERT promoter, shows high levels of the mRNA because the immortalization of the cell line has been carried out by overexpression of hTERT along with E6/E7 viral proteins. From the above investigations, it is evident that mutations present in functionally important part of the proteins indeed lead to drastic changes in their functions which ultimately play a role in carcinogenesis.

### Gene expression of GBM cell lines and comparison with GBM tumor transcriptome

**Figure 6:**
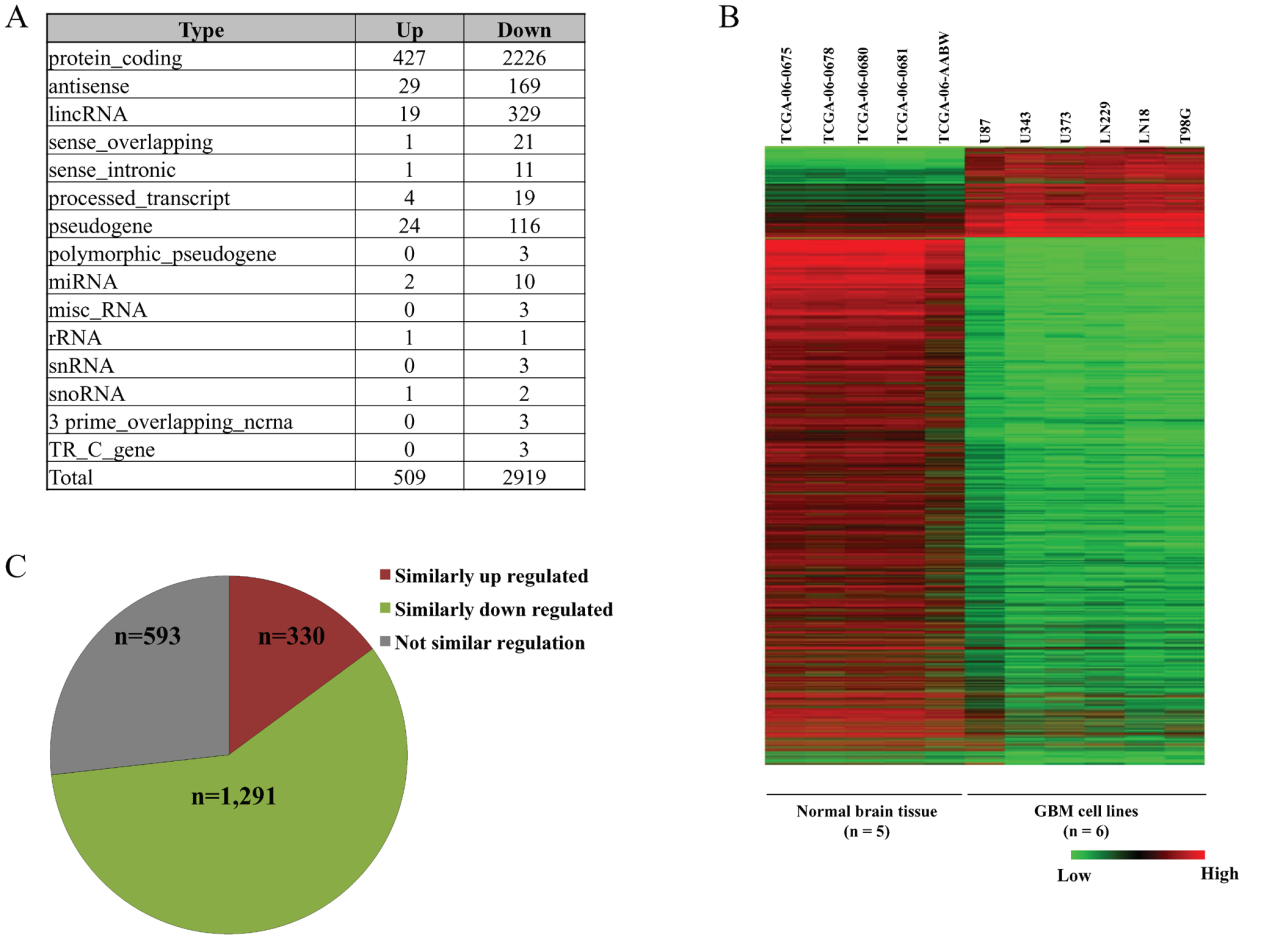
Gene expression analysis of GBM cell lines **A.** Distribution of different classes of RNAs that are differentially regulated across the six GBM cell lines compared to normal brain samples. **B.**. Heat map of the differentially expressed genes in the six GBM cell lines *versus* normal brain samples. **C.** Concordance of the transcriptome data from RNA-seq data of cell lines with Agilent microarray of GBM samples from TCGA. Of the 3,428 differentially regulated genes from cell lines, concordance in 2,214 genes that are also present in Agilent microarray data has been presented.

Differential gene expression profiling was carried out by comparing RNA-seq data of the six cell lines with that of five normal brain tissue samples from TCGA. As per Gencode Version 19 annotation file [55], applying a cut-off of 2 fold change in absolute expression value, we obtained a total of 3,428 differentially regulated genes of which 509 were up regulated and 2,919 were down regulated (Figure 6A and 6B). The differentially expressed genes were classified into the different classes of RNA - protein coding, long non coding RNA and miRNA etc. (Figure 6A) and the entire result was tabulated (Supplementary Table 6).

The differentially regulated genes in the six GBM cell lines were compared with Agilent microarray data of GBM tumor tissue samples from TCGA (Figure 6C). Of the 3,428 genes differentially regulated in the cell lines 2,214 genes (383 up regulated and 1,831 down regulated) were found to be present in Agilent microarray gene list from TCGA GBM tumor samples. Differentially expressed genes from TCGA's Agilent microarray data was selected on the basis of p-value cut-off (p-value ≤ 0.05). A total of 1,621 genes were found to be similarly regulated between in-house cell line data and TCGA GBM Agilent data (Figure 6C) thus giving a concordance of ∼73%.

### Analysis of potential oncogenic gene fusions from RNA-seq data

**Figure 7:**
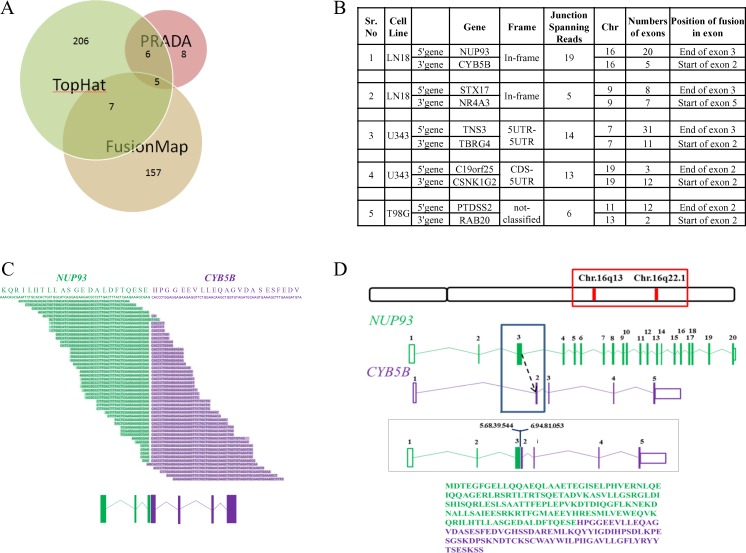
Gene fusion analysis **A.** Number of fusions obtained from each tool- TopHat-Fusion, FusionMap and PRADA. Intersection of each circle represents the fusions found common in between the tools. **B.** Five fusions obtained from all three tools specifying cell line in which it is present, chromosome no., fusion type, total no. of exons for each gene. **C.** Split reads aligning to the gene fusion junction as per TopHat-Fusion prediction. The possible protein sequence present at the junction has been given on top with NUP93 sequence represented in green and CYB5B sequence in purple. The exonic structure of the gene fusion is given at the bottom where boxes represent exons and the lines represent introns. **D.** Chromosomal location of the members of the fusion, exon-intron structure and possible fusion region has been depicted. The entire fusion protein sequence has been provided where amino acids represented by green letters belong to NUP93 and those represented by purple belongs to CYB5B.

Numerous studies discovered gene fusion to be a critical and significant event in hematopoietic tumors [56] as well as solid cancers including GBM [8, 9, 57-62]. In the six cell lines analyzed here, a total of 389 gene fusion events were detected using three different tools: FusionMap [63], TopHat-Fusion [64] and PRADA [65] (Supplementary Table 7), out of which 5 fusions were detected by all three tools (Figure 7A and 7B). A low overlap between the three tools was obtained because PRADA uses stringent criteria for fusion detection and hence only few fusions were obtained from PRADA. Only two of the five fusions namely NUP93-CYB5B and STX17-NR4A3 were found to give rise to in-frame fusion products and both were present in LN18 cell line (Figure 7B).

**Figure 8:**
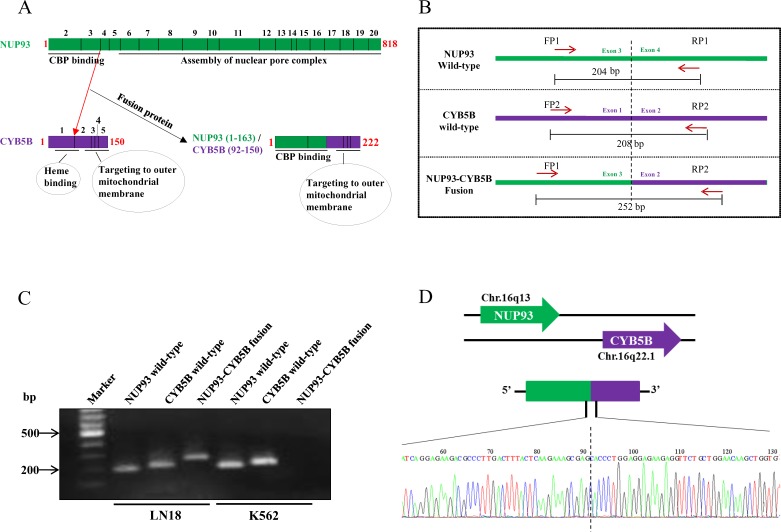
NUP93-CYB5B gene fusion validation **A.** Representative image of the protein structure of wild-type NUP93 and CYB5B and NUP93-CYB5B fusion product. The exons representing each section of the protein sequence has been represented by smaller boxes in the protein schematic and exon numbers (black) have been provided on top of the diagram. The amino acid length of each protein sequences have been denoted in red. **B.** Primer design for detecting wild type and fusion gene for NUP93-CYB5B. FP = Forward primer and RP = Reverse primer. **C.** Semi-quantitative PCR gel picture of amplicons derived from PCR of wild type NUP93 and CYB5B and NUP93-CYB5B fusion products in LN18 and K562. **D.**. Sanger sequencing chromatogram of the fusion junction region.

NUP93-CYB5B gene fusion was taken up for characterization. As per analysis by PRADA, NUP93-CYB5B fusion was represented by a total of 19 junction-spanning reads (Figure 7B). Both the fusion partners are located in the same chromosome (Chromosome 16) where NUP93 has 20 exons and CYB5B has 5 exons (Figure 7B). The predicted reading frame at the breakpoint is shown for NUP93 (green) and CYB5B (purple) fusion (Figure 7C). The predicted intron-exon structure of the fused product shows that the first three exons of NUP93 and last four exons of CYB5B are retained and the fusion is created by joining of the end of exon 3 of NUP93 with the start of exon 2 of CYB5B (Figure 7C and 7D). The genomic breakpoint was mapped to Chr. 16q13 position 56,839,544 for NUP93 and Chr. 16q22.1 position 69,481,053 for CYB5B (Figure 7D). NUP93 is a nucleoporin protein comprising of 818 amino acids and it plays a vital role in nuclear pore complex formation (Figure 8A). While its C-terminal amino acids (∼600) are essential for the assembly of the structural backbone of the nuclear pore complexes [66], the N-terminal 165 amino acids are required for binding to CREB-binding protein (CBP) [67] (Figure 8A). The C-terminal region of CYB5B, a mitochondrial hemoprotein, is essential for its localization to the outer mitochondrial membrane (Figure 8A) [68]. Analysis revealed that the fusion protein encoded by fused transcript is a 222 amino acid protein consisting of the N-terminal 163 amino acids of NUP93 and C-terminal 59 amino acids of CYB5B (Figure 7D and 8A). Thus the fusion protein is likely to target the CBP to the mitochondria with a potential possibility of acetylation of certain proteins located in the mitochondrial outer membrane. Indeed, acetylation of mitochondrial enzymes present in the mitochondrial matrix occurs in nutrient excess condition thus leading to their inactivation [69]. Mitochondrial lysine acetyltransferase has not been identified till date although it has been observed that knockdown of a homolog of histone acetyltransferase in yeast leads to hypoacetylation in mitochondrial enzymes [70]. Acetylation of outer mitochondrial membrane proteins like Voltage-dependent anion channel proteins (VDACs), Fatty acyl-CoA synthetase 1 (ACS-1) and Carnitine palmitoyl-transferase 1 (CPT-1) has been reported to increase their stability while the functional consequence of such acetylation is unknown [71, 72]. Here, we hypothesize that targeting CBP to mitochondria by the fusion protein would result in acetylation and subsequent stabilization of mitochondrial proteins which might provide metabolic advantage for the cancer cell [73, 74].

The primer design for the confirmation of fusion product has been given (Figure 8B). The fusion specific primers have been designed such that the forward primer that can amplify the wild-type NUP93 and the reverse primer that can amplify the wild-type CYB5B can be combined to amplify the fusion product such that the amplicon will be of different size than both the wild-type products (Figure 8B). Semi-quantitative analysis of NUP93-CYB5B fusion revealed the presence of the fusion product in LN18 but not in K562, a human leukemic cell line (Figure 8C). Sanger sequencing validation was carried out for the fusion junction (Figure 8D). The exact fusion junction as predicted by PRADA was confirmed in the Sanger sequencing analysis.

### GBM specific RNA editing events in the cell lines

RNA editing is a molecular process by which RNA sequences are altered post-transcriptionally through base conversion or insertion/deletion. In mammals, especially in humans, the most common type of editing changes includes A to I and C to U base modifications (canonical editing events) carried out by ADAR family of enzymes and APOBEC enzymes respectively [75, 76]. A schematic representation of the pipeline used for detecting potential RNA editing events from RNA-seq data has been provided and the details have been provided in the Materials and Methods section (Supplementary Figure S10). While the average number of editing event per cell line was found to be 18,949, there were a total of 1,04,904 editing events across the cell lines (Supplementary Figure S10).

**Figure 9:**
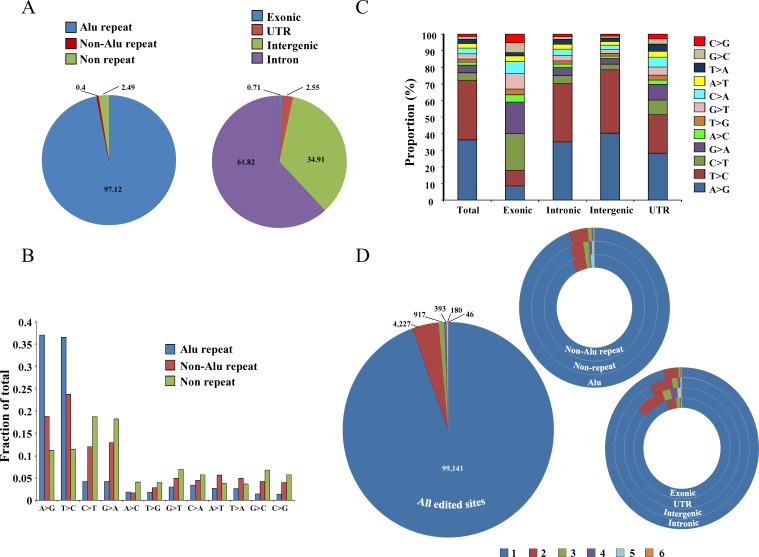
Distribution of editing events across the genome **A.** Distribution of total editing events in different regions of the genome; left panel: in Alu repeat, non-Alu repeat and non-repeat regions and right panel: in exonic, UTR, intergenic and intronic regions. **B.** Distribution of different types of editing events in different regions of the genome (Alu repeat, non-Alu repeat and non-repeat). **C.** Distribution of RNA editing events across different portions of the genome (exonic, intronic, intergenic and UTR). **D.** Recurrence distribution of editing events in the six cell lines in all the edited sites (big circle) or in non-Alu repeat, non-repeat and Alu repeat regions or in exonic, UTR, intergenic and intronic regions (smaller hollow circles); e.g: 99,141 editing events were unique to one sample, 4,227 were in two samples etc. The color code at the bottom represents in how many cell lines an editing event will be present e.g. 1 represents that editing events are present in one of the six cell lines, 2 represents editing events to be present in two cell lines etc.

Majority of RNA editing events, were found in Alu repeat region (97.12%) compared to non-Alu repeat (0.4%) and non-repeat regions (2.49%) (Figure 9A). We also found that the majority of the RNA editing events were present in the intronic regions (61.82%) followed by intergenic (34.91%), UTR (2.55%) and exonic regions (0.71%) (Figue 9A). A total of 75% of the base changes in the Alu repeat regions were of the types A > G and T > C (complementary base change of A > G), which are ADAR specific changes (Figure 9B) [77-79]. However, in non-Alu repeat and non-repeat regions, both ADAR (A > G, T > C) and APOBEC (C > T, G > A) specific RNA editing events were found to occur relatively at higher frequency compared to other editing events (Figure 9B). While the ADAR-specific RNA editing events were seen more in general, it was interesting to note that exonic portions of the genome had higher percentages of APOBEC-specific RNA editing events (Figure 9C). However, other regions like intronic, intergenic and UTR had higher proportion of ADAR-specific RNA editing events (Figure 9C). Low prevalence of Alu repeats in exonic regions explain the reduced ADAR-specific RNA editing events and increased APOBEC-specific RNA editing events. The maximum number of RNA editing events (94.5%) were unique to each cell line while a small proportion of them were found in multiple cell lines (Figure 9D). Among Alu repeat, non-Alu repeat and non-repeat regions, the percentage of unique RNA editing events were more or less similar (∼94%) (Figure 9D). The percentage of recurrent (present in more than one cell line) editing events was maximum in UTR region (13.82%) followed by intergenic (7.81%), exonic (5.36%) and intronic (3.86%) regions (Figure 9D). The list of all the editing events across the cell lines have been tabulated in Supplementary Table 8. Finally, validation by Sanger sequencing was performed and 8 out of 16 selected A to G RNA editing events got validated (Supplementary Figure S7A and S7B).

## DISCUSSION

In this study, we provide a comprehensive characterization of six most widely used GBM cell lines using WES and RNA-seq. Previous studies which characterize glioma cell lines using NGS fall short of comprehensive characterization [10-12]. In the first study, identification of SNVs and structural variations (copy number alterations and gene rearrangments) of one cell line (U87) was carried out from whole genome sequencing (WGS) [10]. In the second study, the authors performed WES of multiple cell lines of different tissue lineages including six GBM cell lines (SF-268, SF-295, SF-539, SNB-19, SNB-75 and U251) and identified indels and cancer-specific SNVs to understand the pharmaco-genomic correlations between specific variants and sensitivity to various anti-cancer drugs [11]. In the third study, characterization of 675 human cancer cell lines was carried out to identify SNVs and gene fusion events from RNA sequencing data [12]. This study included 22 cell lines which were glioma-derived of which three cell lines (T98G, LN229 and LN18) are characterized in the current study. In contrast, we have carried out both WES and RNA-seq of six most commonly used GBM cell lines (T98G, LN229, LN18, U87, U343 and U373). Further, an in-depth data analysis was performed to find overall genetic alterations that included SNVs, indels, transcriptome changes, gene fusions and RNA editing events. Moreover, we have validated various genetic alterations (SNVs, indels, gene fusion and RNA editing events) through Sanger sequencing and quantitative PCR based methods.

We particularly focus on the genetic alterations in cell lines which have potential roles in carcinogenesis. From the total SNV data, we have carried out analysis to unearth cancer-specific alterations. Further, we compared the mutation spectrum of GBM cell lines with that of GBM tissue samples reported by other groups [8, 9] and this revealed various genes altered in GBM scenario to be altered in these cell lines also. Although Klijn et al. have uncovered SNVs from RNA-seq data, analysis of SNVs from whole exome sequencing data will be more comprehensive as it will cover vast majority of all the protein coding genes. SNV data of genes not expressed or expressed at low levels in RNA copy numbers will be missed out from RNA-seq data. We have also carried out indel analysis from WES. Functional importance of genes altered by mutation like TP53, MLL3, BRCA2, NF1 etc. has been investigated. Important emphasis has also been given to hTERT promoter mutation status through Sanger sequencing as this particular mutation is predominant among GBM patients. We have checked the gene expression levels in GBM cell lines with respect to normal brain samples and we have found good concordance with GBM tumor tissue microarray data. Moreover, we also analyzed both RNA-seq and WES data to find out potential RNA editing events. Further, we compared these editing events with normal brain editome data to find out editing events occurring in diseased condition. To our knowledge, such a comprehensive study of genetic alterations in GBM cell lines has not been carried out till now.

Among indels and cancer-specific SNVs reported previously to be present in GBM tumor tissues [8, 9], the most frequent alterations were observed in TP53, PTEN, TCHH and MLL3. Further, mutations in EGFR, NF1and PDGFRA were found in some of the cell lines. Chromatin modifier genes like MLL3, ATRX, MLL4, SETD2 and SRCAP were seen to be mutated. The six GBM cell lines were seen to harbor mutations in 19 DNA repair related genes. However, none of the genes in BER or MMR pathways were observed to be mutated in any of the cell lines. Global gene expression profiling followed by pathway analysis of p53 mutant vs p53 wild-type cell lines revealed p53 regulated processes like cell differentiation, neuron development, cell adhesion, cell proliferation and apoptosis to be significantly enriched. Interestingly, none of the cell lines showed mutation in IDH1 gene. Our study also revealed that U87, T98G, LN229, U343, U251, and U373 cell lines harbor promoter activating mutations in the hTERT genes with a concomitant up regulation of hTERT transcript levels. No mutation was found in LN18 and SVG and hTERT mRNA levels were also found to be low in these cell lines. A comparison of the differentially regulated genes in the cell lines with that of GBM tumor transcriptome data derived from TCGA gave a concordance of ∼73% suggesting that the transcriptomic make up of these cell lines closely resemble that of GBM tumor tissues. A total of over three hundred gene fusion events were identified in the six cell lines using three different tools and five such fusions came up to be common in all three tools. Two gene fusions, NUP93-CYB5B and STX17-NR4A3, were in-frame fusions both of which were present in LN18 cell line. Validation of NUP93-CYB5B fusion using Sanger sequencing revealed the exact fusion junction as predicted by PRADA. The targeting of CBP to the mitochondrial outer membrane by NUP93-CYB5B fusion protein is likely to result in the acetylation of proteins like VDACs, ACS-1 and CPT-1 which may provide metabolic advantage to the cells. While ACS1 and CPT1 are involved in fatty acid metabolism and transport into mitochondria respectively, VDACs help in ATP transport out of mitochondria [73, 80]. Further, β-oxidation of fatty acids in the mitochondria results in increased energy production in the cancer cells with the resultant increased proliferation [73]. Indeed, inhibition of fatty acid synthesis or its transport into mitochondria results in growth inhibition and increased apoptosis [73, 74]. Hence, we predict that CBP-mediated acetylation of these proteins with resultant stabilization is likely to provide survival advantage to the cancer cells. Further, RNA editome analysis revealed numerous RNA editing events across the cell lines. As observed by others, we found majority of RNA editing events in Alu repeat region present both in intronic and intergenic regions, while its significance is not known yet [81]. We also found significant RNA editing events in the UTR region, in particular 3′UTR, which is likely to alter miRNA binding leading to the alteration of the gene expression pattern which may contribute to transformation and other cancer cell properties [82].

Thus, a comprehensive alteration landscape that includes cancer specific SNVs, indels, transcriptome profile, gene fusions and RNA editing events is generated from whole exome and RNA sequencing data for six GBM cell lines. Since these cell lines are routinely used for *in vitro* and *in vivo* studies by glioma biologists, our study would be of great help to the scientific community.

## MATERIALS AND METHODS

### Cell lines used

The cell lines used are glioblastoma (GBM) cell lines U87, U343, U251, U373, LN18, LN229 and T98G and immortalized human astrocytic cell lines SVG [83] and IHA (NHA-E6/E7-hTERT) [84]. U343, LN18, IHA and SVG were obtained from the laboratory of Dr. A. Guha, University of Toronto, Canada. U87, T98G, U251, LN229 and U373 were obtained from Sigma Aldrich (Saint Louis, Missouri, USA). All cell lines were cultured in Dulbecco's Modified Eagles' Medium containing 10% Fetal Bovine Serum at 37°C and 5% CO_2_.

### DNA isolation, quantification and library preparation for whole exome sequencing

DNA was isolated from 5 million cells for each cell line using Qiagen DNA Minikit (Catalog no. 51306) for obtaining good quality protein and RNA free genomic DNA. The concentration of DNA was determined using Quant-iT™ PicoGreen^®^ dsDNA Assay Kit (Catalog no. P11496) where the standard curve was obtained from increasing dilutions of lambda DNA (Stock = 500 ng/μl). DNA library was prepared using TruSeq Library Preparation kit (Catalog no. RS-122-2001) as per manufacturer's guidelines. DNA was sheared using Covaris sonicator (S220) and the library was prepared. The obtained DNA library was quantified using Agilent's Biolanalyzer. Next, DNA libraries (one for each sample) were taken in batches of six and pooled together for exome enrichment using Truseq Exome Enrichment kit (Catalog no. FC-121-1008). Finally the exome libraries were quantified by real-time qPCR using Illumina adapter specific primers for library quantification.

### RNA isolation, quantification and library preparation for whole RNA sequencing

Cells were harvested using Tri Reagent^®^(Sigma, Catalog no. T9424) and RNA were extracted using the standard Chloroform-Isopropanol method. The RNA samples were checked for quality and quantity using Agilent's Bioanalyzer. The library for sequencing was prepared using TrueSeq RNA sample preparation kit as per the manufacturer's guidelines (Catalog no. RS-122-2001). The library was then quantified using Agilent's Bioanalyzer as well as real-time qPCR.

### Cluster generation and sequencing

10 picomoles of each pooled exome or RNA library was taken, the strands were denatured and finally subjected to cluster generation on the flow-cell in the c-Bot system using TruSeq PE Cluster kit (Catalog no. PE-401-3001). The flow cell was finally subjected to two rounds of sequencing (for Read1 and Read2) and the results were obtained as intensity files. Sequencing was conducted on Illumina HiScanSQ using Truseq SBS V3 technology for 100 and 50 base pair paired-end reads for exome and RNA sequencing respectively (Catalog nos. FC-401-3001 and FC-401-3002 respectively).

### cDNA conversion

For cDNA conversion, 2 μg good quality RNA was used per reaction. Applied Biosystems™ High Capacity cDNA Reverse Transcription kit (Part no. 4368813) was used. The cDNA strand synthesis was carried out in Biorad S1000™ Thermal Cycler.

### PCR amplification and Sanger sequencing

Genomic DNA or cDNA was taken as template for PCR amplification. Thermo Scientific's DyNAmo (Catalog no. F-416) reagent was used as amplification buffer along with primer of interest. Biorad S1000™ Thermal Cycler was used for PCR amplification.

Primer sequences have been provided in Supplementary Table S9. A schematic representation of the PCR strategy used for amplification of the promoter region of hTERT has been given (Supplementary Figure S8).

### Real-time qPCR quantification

Thermo Scientific's DyNAmo (Catalog no. F-416) reagent was used for this purpose with cDNA from good quality RNA used as template. Applied Biosystems™ 7900HT Fast Real-Time PCR system was used. GAPDH was used as internal control. Primer sequences have been provided in Supplementary Table S9.

### Data analysis pipelines

#### Whole exome sequencing analysis

We have carried out 100 bp paired-end sequencing for this purpose.

#### Alignment and Recalibration

The sequencing output in the form of base intensity files was converted to fastq format and subsequently de-multiplexed using bcl2fastq [85]. Next, BWA or Burrows Wheeler Aligner version 0.6.2 was used to align the reads to the human reference genome hg19 i.e., Human Genome Reference Consortium build 37 (GRCh37) [86]. Post-alignment, we obtained the ‘.sam’ file which was converted to binary format or ‘.bam’ files using Samtools 0.1.18 [87]. For co-ordinate sorting and duplicate removal, Picard 1.73 was used [88]. Read re-alignment, required for properly calling indels, was done using GATK 2.7-2 module IndelRealigner [89]. The same tool's module called BaseRecalibrator was used for base recalibration.

#### Variant Calling

The detection of Single nucleotide variants (SNVs) and indels (small insertions/deletions) was carried out using GATK's module called UnifiedGenotyper [89]. Finally, variant annotation was done using Oncotator v.4.2.2 [17].

#### Filtering for Cancer-specific SNVs

To filter out cancer-specific SNVs from total SNVs, the SNVs obtained from GATK UnifiedGenotyper was passed through stringent filteration steps (Supplementary Figure S9). SNVs with ESP6500 frequency ≥ 0.00009 were removed as SNVs having no disease consequences [90]. Similarly, in the next step, SNVs with 1000 genomes database frequency ≥ 0.0005 were eliminated [91]. The SNVs obtained at this step were divided into those reported in COSMIC database *versus* those not reported in the same [18]. The SNVs present in COSMIC were considered as single nucleotide changes that may have possible role in carcinogenesis and hence cancer-specific SNVs. Those SNVs not present in COSMIC were compared with the SNVs obtained from GBM TCGA exome sequencing data [8, 9]. Those SNVs present in the TCGA data were taken to be cancer-specific changes while SNVs not present in the above database were taken up for the following steps of filtration. SNVs with data set frequency, i.e. frequency of occurrence among the samples tested, ≥ 0.5 were eliminated. Finally the above SNVs were compared with dbSNP build 137 to remove potential single nucleotide polymorphisms [92]. Hence, all mutations obtained from the previous step, those obtained as present in COSMIC database and those which came up as already reported GBM-specific changes as per TCGA were combined to form the cancer-specific SNVs set. The rest of the SNVs were considered as non-specific.

### RNA sequencing analysis

We have carried out 50 bp paired-end sequencing for this purpose.

#### Transcriptome analysis

The whole RNA sequencing data was aligned using PRADA tool [65]. Duplicate removal was carried out using Picard 1.73 [88]. The RNA-seq reads were counted over gene exons using HtSeq [93]. Genes were annotated as per Gencode Version 19 annotation file [55]. We used the DESeq size factor correction to account for differences in sequencing depth between the samples. For differentially expressed gene identification between GBM cell lines compared to normal brain tissue samples (from TCGA), we used DESeq with p-adjusted cut-off of 0.05 [94].

#### Gene fusion analysis

Analysis of potential gene fusions in each cell line from RNA sequencing data was carried out using three different tools - PRADA, FusionMap and TopHat-Fusion. 1) PRADA: Pipeline for RNA sequencing Data Analysis [65]. PRADA aligns RNA sequencing reads to a composite reference database comprising of whole genome reference sequence (hg19) and reference transcriptome sequence (Ensembl64) using BWA. To filter out potential gene fusions, the following criteria were used- -mm 3 -junL 40 -minmapq 30. 2) FusionMap: RNA-seq reads for each cell line was aligned using PRADA-Preprocess-bi tool and the aligned reads were then fed into FusionMap [63]. The following parameters were used for fusion calling - MinimalFusionAlignmentLength = 25 FusionReportCutoff = 1 NonCanonicalSpliceJunctionPenalty = 2 MinimalHit = 2 MinimalRescuedReadNumber = 1. 3) TopHat-Fusion: The RNA-seq data was mapped using TopHat2 with the following options -r 0 **-**p 14 -no-coverage-search -mate-std-dev 80 -max-intron-length 100000 -fusion-min-dist 100000 -fusion-anchor-length 13 -fusion-search -keep-fasta-order -bowtie1. Using the mapped RNA-seq data, fusion transcript candidates were filtered by tophat-fusion-post [64].

#### RNA editing events

RNA-seq data from each cell line was used to identify RNA editing events following a rigorous and robust pipeline (Supplementary Figure S10) [95, 96]. The RNA-seq reads were aligned to reference genome (hg19) and transcriptome (Ensembl64) using PRADA-Preprocess-bi tool. Next, variants were called using GATK's UnifiedGenotyper with options stand_call_conf of 0 and stand_emit_conf of 0 [89]. The total variants obtained were then filtered to remove potential polymorphisms by comparing with dbSNP [92], 1000 genomes database [91] and ESP6500 [90]. First six bases of each read were discarded to remove artificial mismatches caused by random-hexamer priming. The editing events present in Alu regions were separated out. The editing events present in non-Alu regions were then subjected to further steps to filter out spurious changes- 1) each editing event were to be represented by atleast 3 reads containing altered nucleotide with a minimum frequency of altered nucleotide being 0.1; 2) any site present in simple repeats were removed; 3) any candidate change present within 4 bps of known splice junction were removed; 4) sites present in homopolymer runs of ≥ 5 bps were removed; and, 5) sites located in regions having high similarity to sequences present in other parts of the genome (found out using BLAT) were removed. Editing events present in non-Alu regions along with those found in the Alu regions were compared with normal brain editome data to eliminate editing events which have no carcinogenic consequences [96]. Events obtained from above step were compared with SNVs obtained from whole exome sequencing data of each corresponding sample to remove RNA editing events that are wrongly called due to presence of sample-specific genetic variations. This gives GBM cell-line specific RNA editing events.

## SUPPLEMENTARY MATERIAL FIGURES AND TABLES





















## Abbreviations

GBM: glioblastoma
SNP: Single Nucleotide Polymorphism
SNV: Single Nucleotide Variation
indels: insertions and deletions
NGS: Next Generation Sequencing
WES: Whole Exome Sequencing
RNA seq: RNA Sequencing
TCGA: The Cancer Genome Atlas
CCLE: Cancer Cell Line Encyclopedia
COSMIC: Catalogue Of Somatic Mutations In Cancer
IHA: Immortalized Human Astrocytes
