# Supplementary material for: Elucidating the cancer-specific genetic alteration spectrum of glioblastoma derived cell lines from whole exome and RNA sequencing
Source: Oncotarget. 2015 Oct 19;6(41):43452–71. doi: 10.18632/oncotarget.6171 (PMC4791243; doi:10.18632/oncotarget.6171)
Supplement: Supplementary file 1 [file oncotarget-06-43452-s001.pdf]

Elucidating the cancer-specific genetic alteration spectrum of glioblastoma derived cell lines from whole exome and RNA sequencing

Supplementary Material

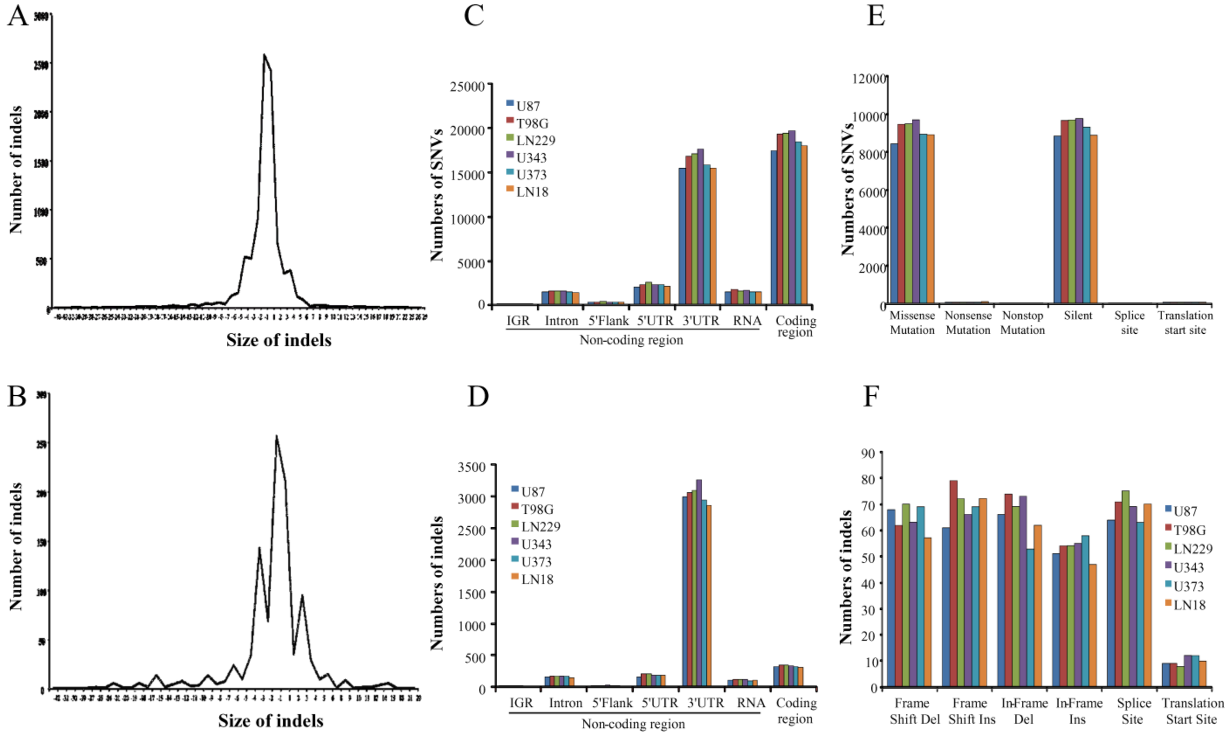

Supplementary Figure S1: Indel Power law distribution and functional classification of single nucleotide variants and indels. The number of indels was plotted on Y-axis and the size of indels plotted along the X-axis. Indel distribution plot for all indels called across the six cell lines **(A)** and indel distribution plot for indels present in the coding regions **(B).** The number of SNVs and indels are plotted in the Y-axis- **C.** Distribution of SNVs according to genomic location. **D.** Distribution of indels according to genomic location. **E.** Distribution of coding SNVs according to the change in protein it may cause. **F.** Distribution of coding indels according to the change in protein it may cause.

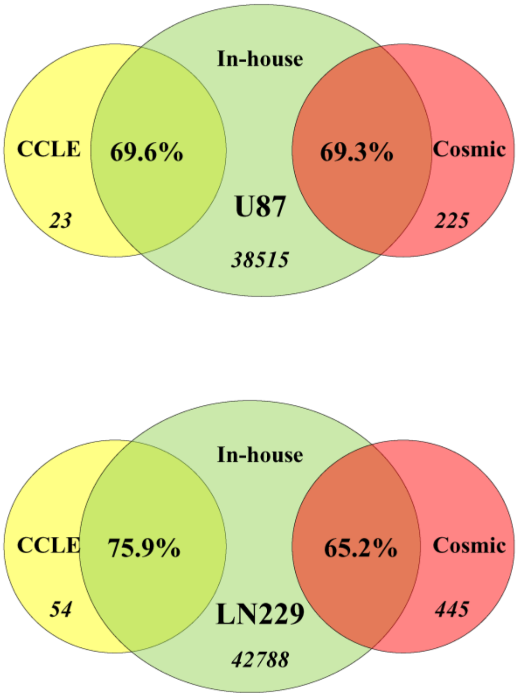

Supplementary Figure S2: Single nucleotide variation comparison with available datasets. The green circles represent lab data, the yellow and the red circles represent CCLE and Cosmic data respectively. The percentage of concordance for SNVs per cell line has been represented by the number in the intersection of each circle. The percentages given here are calculated with respect to the total SNVs present in CCLE or Cosmic databases. The total number of SNVs present in each dataset has been provided in italicized numbers.

Gene name	Chromosome	Start position	Reference Allele	Tumor Allele	Cell Lines	Sanger sequencing status
TP53	17	7577120	C	T	U373	Confirmed
EGFR	7	55229216	C	T	U373	Confirmed
PDGFRA	4	55131140	G	T	U343	Not confirmed
FAT2	5	150945921	C	T	U343	Confirmed
SETD2	3	47165691	C	A	T98G	Confirmed
SRCAP	16	30749088	C	G	T98G	Confirmed
PTEN	10	89717695	C	CTT	U373	Confirmed
NF1	17	29553443	A	AC	U373	Confirmed

Supplementary Figure S3. Sanger sequencing validation of selected novel SNVs and indels.

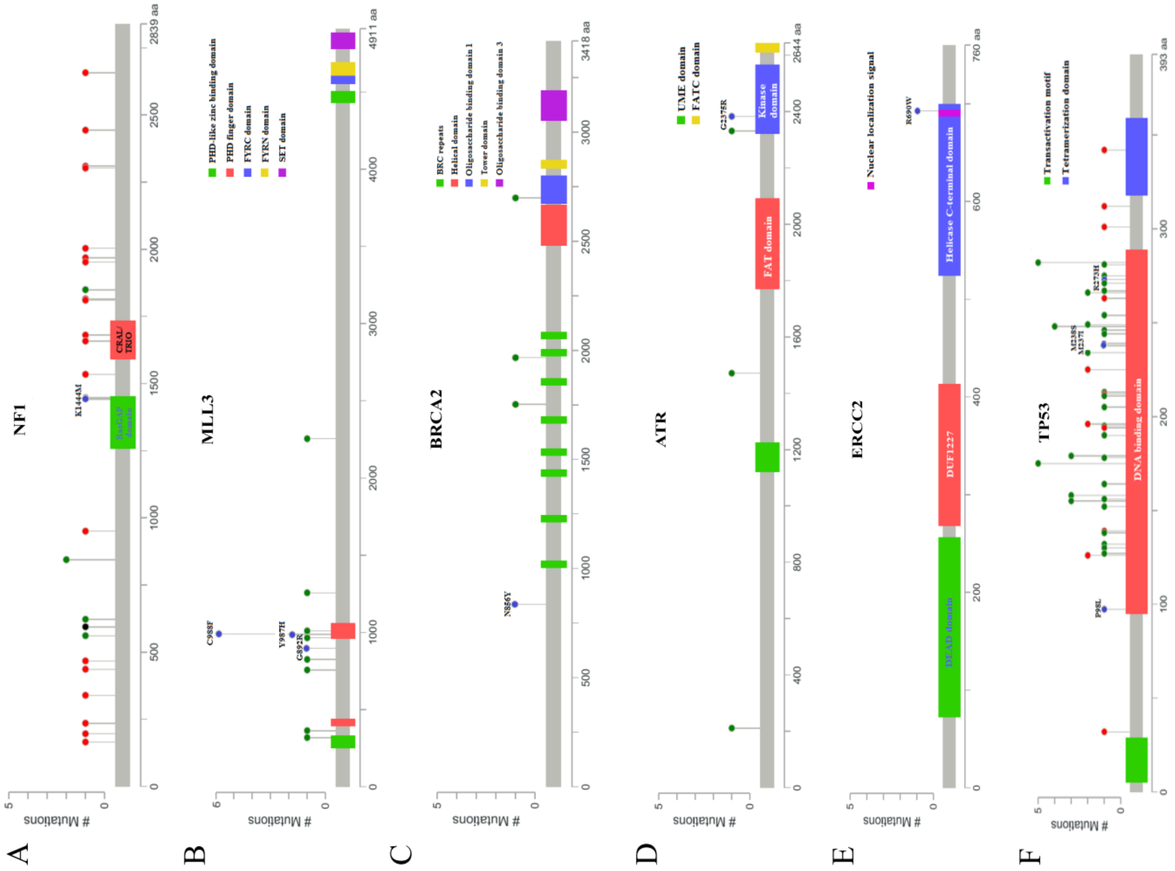

Supplementary Figure S4. Protein domain structure and location of mutation. The protein domain structure as per cBioPortal [97] is given for each gene investigated - **A.** NF1, **B.** MLL3, **C.** BRCA2, **D.** ATR, **E.** ERCC2 and **F.** TP53. The X-axis represents the number of samples in which the mutation at a position is present. Red and green dots indicate positions of nonsense/splice-site/frame-shift and missense mutations respectively present in TCGA GBM tumor tissue samples [9]. Blue dots indicate mutation found in GBM cell lines in this study. The amino acid change for mutation found in cell lines has been provided.

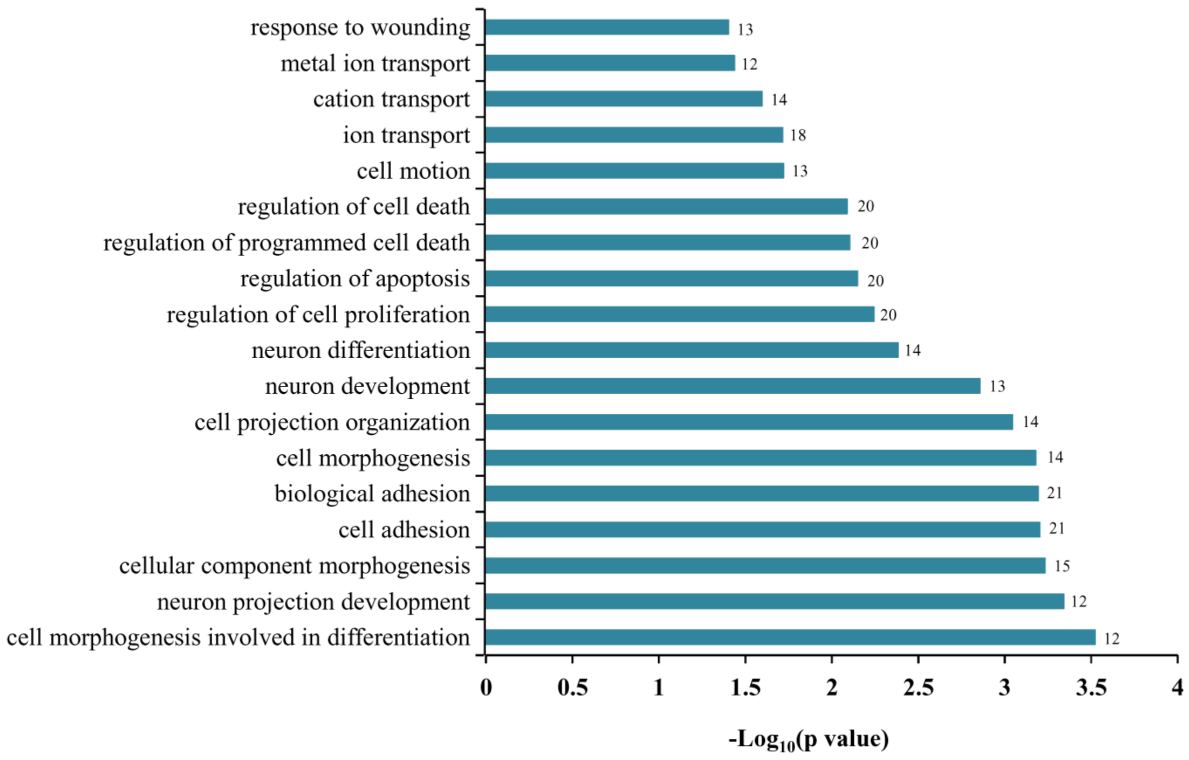

Supplementary Figure S5: DAVID Gene Ontology (GO) Analysis of genes differentially expressed genes in TP53 mutant versus TP53 wild type cell lines. Pathways with p value < 0.05 and >10 enriched genes were selected. The biological processes are given in the Y axis while the negative of Log_{10} of the p value is given in the X axis. The number of enriched genes for each biological process has been provided on the right hand side of each bar.

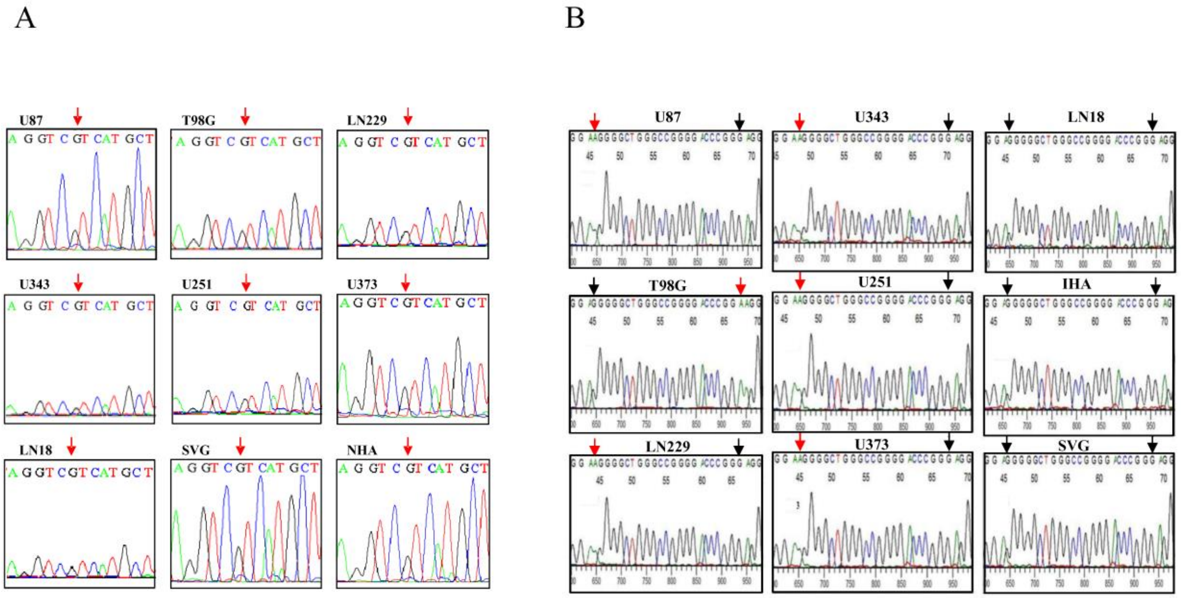

Supplementary Figure S6: Sanger sequencing analysis of IDH1 mutation and hTERT promoter mutation in GBM cell lines. A. Chromatogram for IDH1 R132 mutation. Arrow indicates the base that undergoes alteration, the wild-type base being Guanine and the mutant base is Adenine. Note that no mutation was identified in IDH1 in any of the cell lines. **B.** Chromatogram for hTERT promoter mutations. In each cell line chromatogram, left side arrow refer to 1, 295,228 position and right side arrow refers to 1,295,250 position. Red arrow indicates mutant base (Adenine) whereas, black arrow indicates no mutation (Guanine).

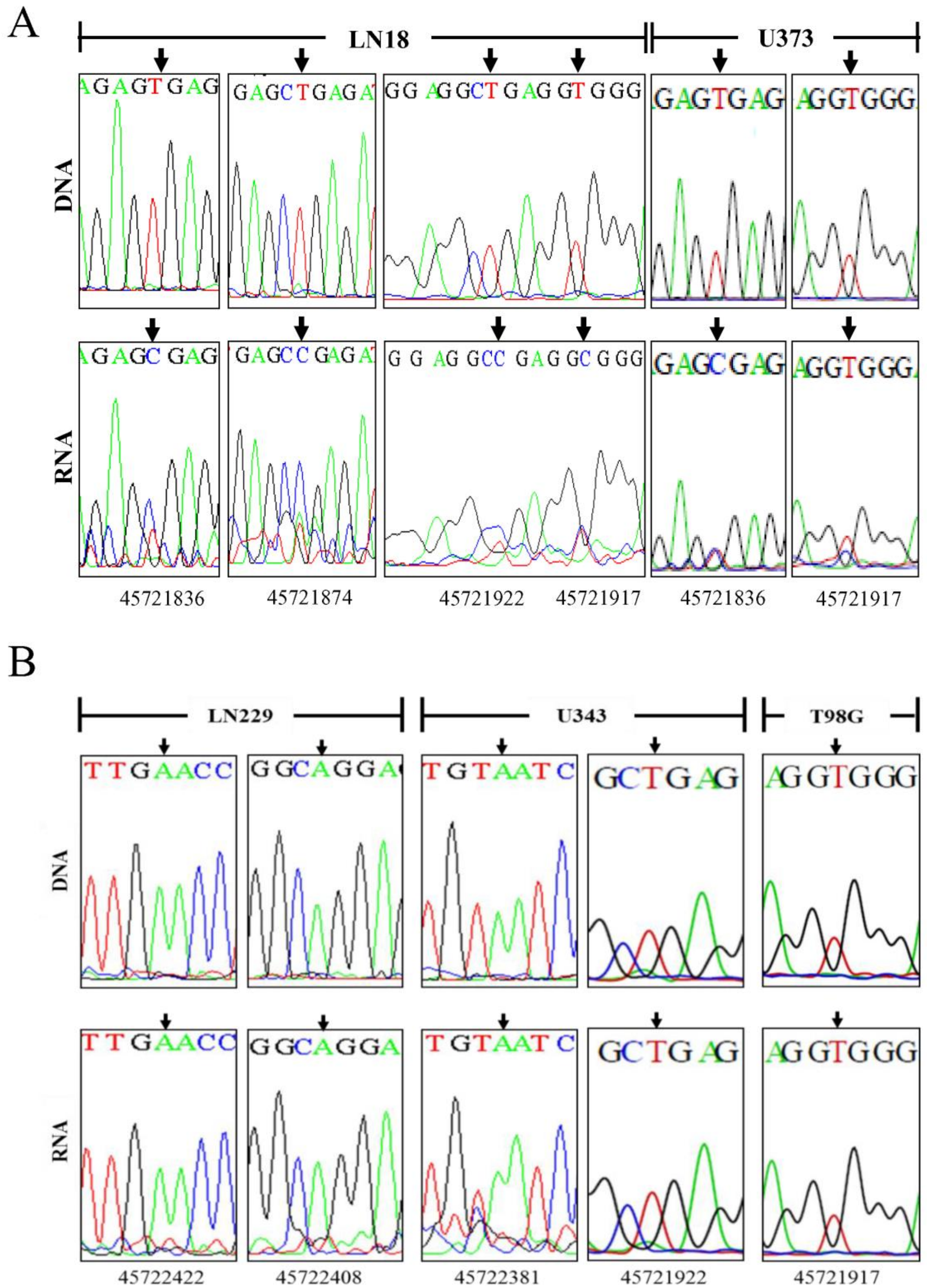

Supplementary Figure S7: RNA editing validation. Representative images of sites subjected to validation by Sanger sequencing**.** Arrow indicates position of potential RNA editing event as given by RNA sequencing analysis. The genomic co-ordinate for the position has been provided at the bottom. **A.** Positions validated by Sanger sequencing. **B.** Positions not validated by Sanger sequencing.

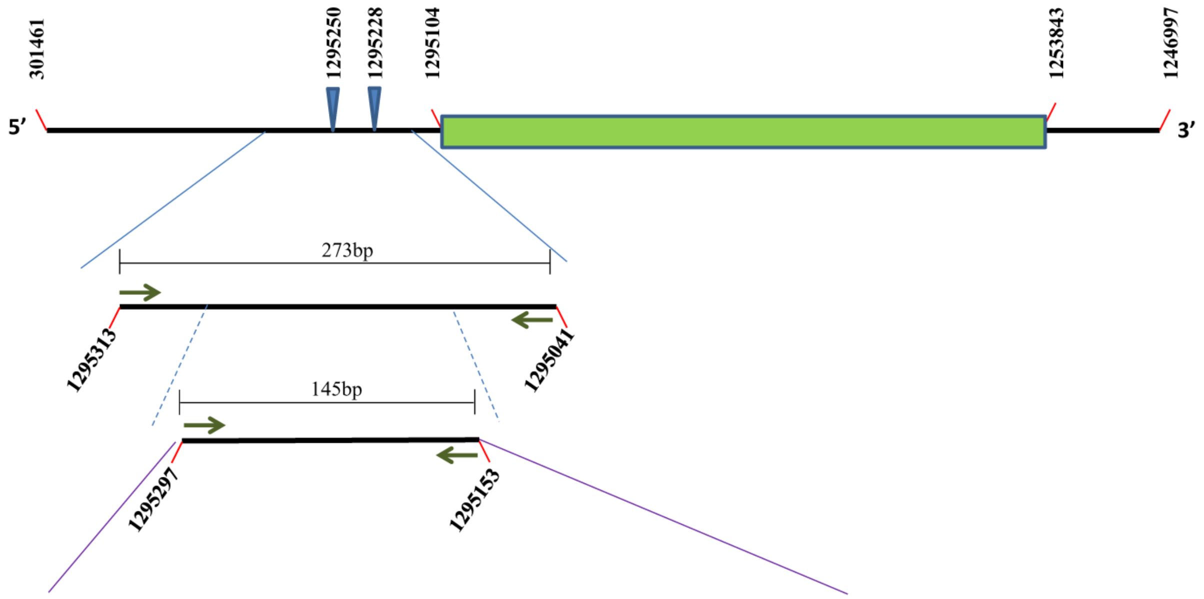

5'GCGCTGCCTGAAACTCGCGCCGCGAGGAGAGGGCGGGGCCGCGGAAA
GGAAGGGGAGGGGCTGGGAGGGCCCGGA**G**GGGGCTGGGCCGGGGACC
CGG**G**AGGGGTCGGGACGGGGCGGGGTCCGCGCGGAGGAGGCGGAGCT
GGAA **3'**

Supplementary Figure S8: Primer designing strategy [53]. Nested PCR strategy used to amplify hTERT promoter region for Sanger sequencing. Green box represents the open reading frame. To the left of the ORF, the promoter sequence will be present. The blue triangles represent the two positions where C>T mutation causes promoter activating changes. The first pair of primers amplifies a 273 bp product that contains the two mutation sites. Region interior to the above amplified sequence will be amplified by the second set of primers to give a 145 bp product. Sequence of the promoter region amplified for sequencing (sequence provided as per amplification by reverse primer) has been provided. The red colored G residues will be altered to A in case of mutants.

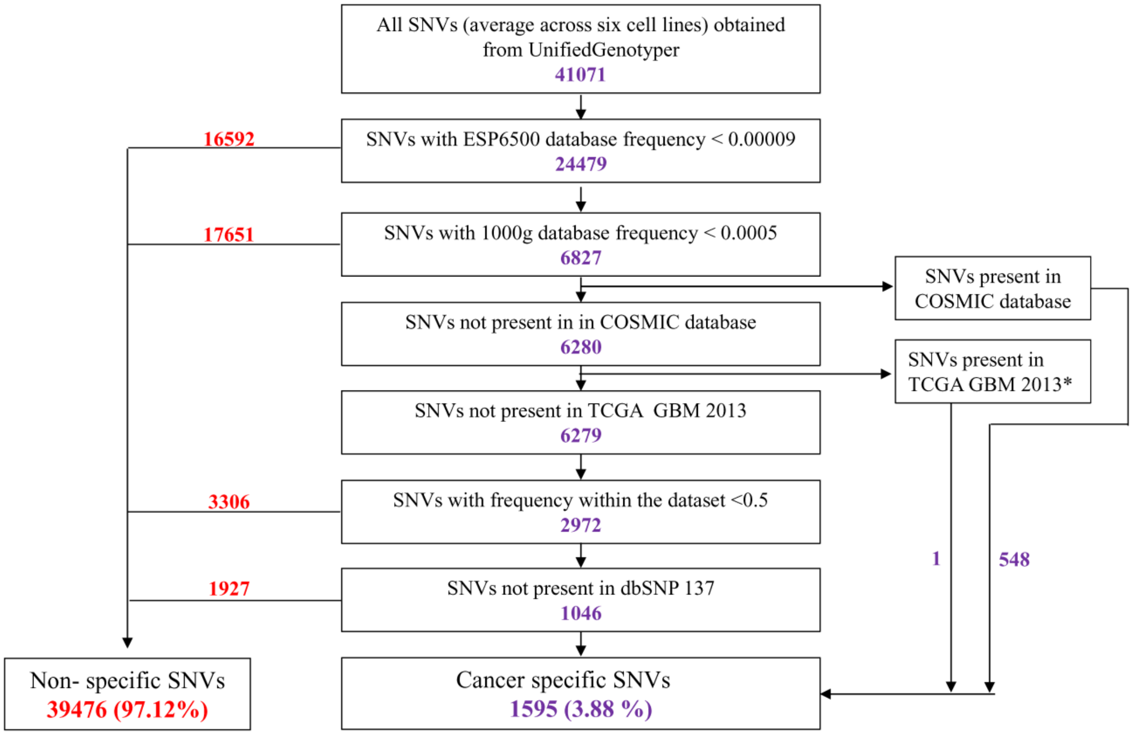

Supplementary Figure S9: Analysis pipeline for filtering out cancer-specific SNVs. Each step for filtering out cancer-specific SNVs has been summarized. The purple numbers represent the SNVs included to be potential cancer-specific in each step and the numbers given in red color represent the SNVs eliminated in each step as non-specific. The percentage of cancer-specific versus non-specific SNVs has been provided in the brackets.

*TCGA GBM 2013 refers to SNVs obtained as important for GBM progression [9].

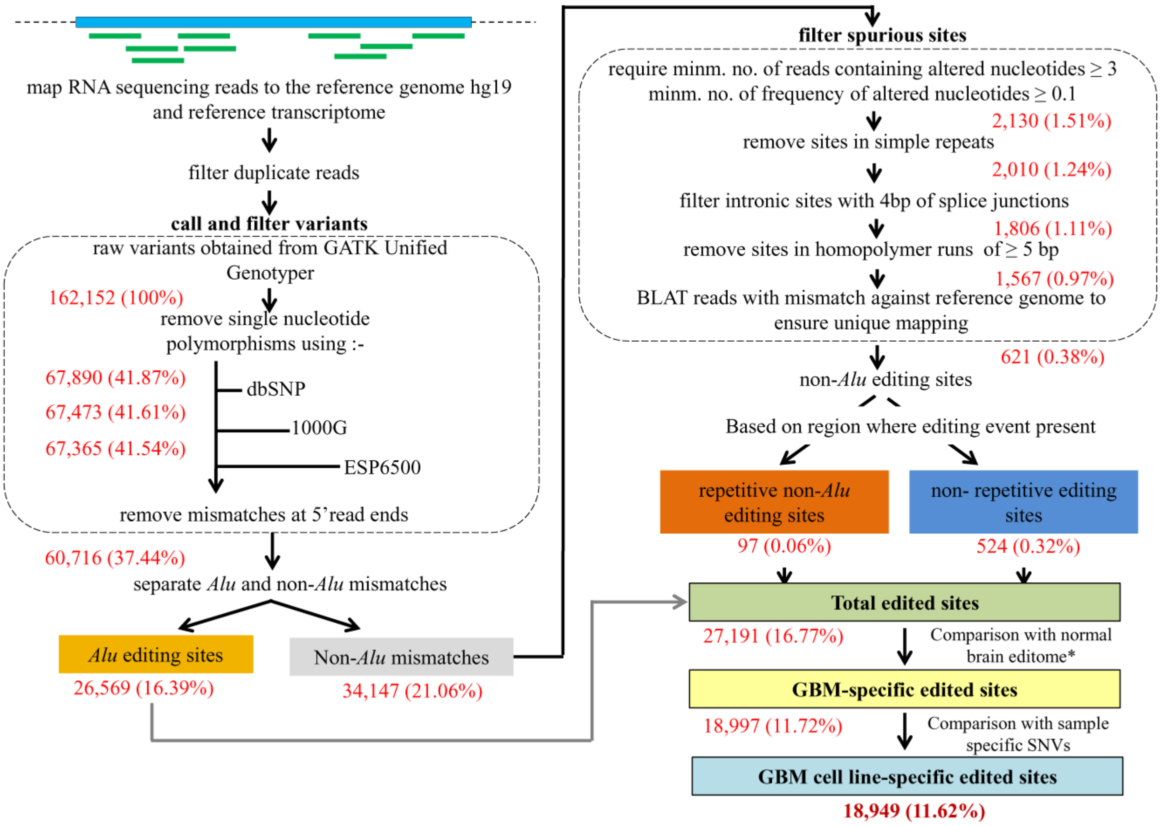

Supplementary Figure S10: Analysis pipeline for filtering out RNA editing events from RNAseq data. Summary of different filtration criteria used to find out RNA editing events. In each step, the number of variants remaining after applying filtration criteria has been given in red font. The percentages in brackets denote the percentage of variants filtered as compared to the total variants called by UnifiedGenotyper.

*Normal brain editome was obtained from Ramaswamy *et al*., 2013 [9].
